# miRNomic Signature in Very Low Birth-Weight Neonates Discriminates Late-Onset Gram-Positive Sepsis from Controls

**DOI:** 10.3390/diagnostics11081389

**Published:** 2021-07-31

**Authors:** Eva Serna, Anna Parra-Llorca, Joaquín Panadero, Máximo Vento, María Cernada

**Affiliations:** 1 Department of Physiology, Faculty of Medicine, University of Valencia, 46010 Valencia, Spain; 2 Division of Neonatology, University & Polytechnic Hospital La Fe, 46026 Valencia, Spain; annaparrallorca@gmail.com (A.P.-L.); Maximo.Vento@uv.es (M.V.); 3 Health Research Institute, University & Polytechnic Hospital La Fe, 46026 Valencia, Spain; 4 Igenomix R&D, 46980 Valencia, Spain; joaquin.panadero@igenomix.com

**Keywords:** neonatal sepsis, miRNomic signature, very low birth-weight neonates, late-onset Gram-positive sepsis

## Abstract

Background and Objectives. Neonatal sepsis is a serious condition with a high rate of mortality and morbidity. Currently, the gold standard for sepsis diagnosis is a positive blood culture, which takes 48–72 h to yield results. We hypothesized that identifying differentially expressed miRNA pattern in neonates with late-onset Gram-positive sepsis would help with an earlier diagnosis and therapy. Methods. This is a prospective observational study in newborn infants with late-onset Gram positive bacterial sepsis and non-septic controls. Complementary to blood culture, an aliquot of 0.5 mL of blood was used to determine small non-coding RNA expression profiling using the GeneChip miRNA 4.0 Array. Results. A total of 11 very low birth-weight neonates with late-onset Gram-positive sepsis and 16 controls were analyzed. Further, 217 differentially expressed miRNAs were obtained between both groups. Subsequently, a combined analysis was performed with these miRNAs and 4297 differentially expressed genes. We identified 33 miRNAs that regulate our mRNAs, and the most relevant biological processes are associated with the immune system and the inflammatory response. Conclusions. The miRNA profiling in very low birth-weight neonates distinguishes late-onset Gram-positive sepsis versus control neonates.

## 1. Introduction

Neonatal sepsis is a serious systemic condition caused by bacteria, virus, or fungus and characterized by hemodynamic changes resulting in great mortality and short- and long-term morbidities in survivors [1]. Neonatal sepsis is responsible for up to 20% of all deaths in very low birth-weight (VLBW) infants. Moreover, VLBW infants with sepsis are nearly three times more likely to die and/or develop cerebral palsy and neurodevelopmental impairment [2]. From a pathophysiological point of view, neonatal sepsis has been divided into early-onset sepsis (EOS), defined as sepsis occurring within less than 72 h after birth, and late-onset sepsis (LOS), occurring thereafter. In high income countries, the incidence of EOS in VLBW amounts to 1.38 per 1000 live births infants and 11.9% for LOS.

Clinical signs and symptoms in newborn sepsis are quite unspecific. Blood culture is the gold standard for sepsis diagnosis. However, it frequently yields false negative results either owing to a small sample volume and/or the administration of antibiotics to the mother prior to delivery. Moreover, reliable results are often not available before 48–72 h, thus delaying therapeutic decisions [3,4,5]. To overcome these difficulties, alternative predictors of newborn sepsis have been consistently sought; however, none of the currently available biomarkers have provided clinicians with rapid, highly specific, and sensitive results [6]. Hence, although C-reactive protein, Procalcitonin, or Interleukin-6 in combination have been reported as reliable biomarkers, none of these have shown sufficient sensitivity and specificity [7,8,9,10,11]. Recent studies using tumor necrosis factor-alpha (TNF-alpha) [12], neutrophil CD64 [13], or toll-like receptors (TLR) [14] as early diagnostic markers have added little to the armamentarium of sepsis diagnosis.

MicroRNAs (miRNAs) are specialized short non-coding RNAs (20–22 nt) that inhibit target mRNA translation and are involved in numerous mammalian processes that are essential for development and survival [15]. Moreover, circulating miRNAs have also been employed as biomarkers of diseases [16,17].

Panels of miRNAs have been described in patients with inflammatory and/or infectious diseases, suggesting that circulating miRNAs may also be suitable biomarkers for sepsis. Most of these studies have been carried out in the adult population [18], while experience in neonatal sepsis is still very limited [19,20,21,22]. Chen et al. reported an over-expression of miRNA-101 and miRNA-185 and an under-expression of miRNA-29a, miRNA-141, miRNA-96, miRNA-181a, and miRNA-1184 in septic neonates compared with non-infected controls [23]. Li et al. reported an under-expression of mi-RNA129-5p in a model induced with LPS that caused a more severe increase of TNF-α and IL-8 in neonatal sepsis [24]. Furthermore, a study focused on specific miRNA such as miRNA 15b and miRNA 378a found that both were able to discriminate sepsis and to correlate with CRP or respiratory rate. Moreover, Yu HR et al. found that miRNAs have the potential to be useful therapeutic targets for certain infectious or inflammatory conditions by modifying the neonatal immune system and increasing the production of TLR [25].

In the present study, we have analyzed the miRNome in VLBW with LOS, aiming to identify a differentially expressed miRNA pattern as compared with non-septic controls that would allow a rapid and specific diagnosis and establishing a suitable treatment.

## 2. Materials and Methods

### 2.1. The Study Design and Patients’ Characteristics

This is a prospective, observational, case-control study performed in the Division of Neonatology of the University and Polytechnic Hospital La Fe (HUiP) (Valencia, Spain). The study was approved by the Scientific and Ethics Committee for Biomedical Research (CEIm) (2019/0195 and 2017/0183).

Inclusion criteria included a study cohort of VLBW infants with positive blood culture and diagnosis of LOS to Gram-positive bacteria. The control cohort consisted of non-infected VLBW matched for birth weight and gestational age and with a similar clinical status. Parents signed an informed consent form. Sepsis was considered when one risk factor or three or more of the clinical signs or symptoms were present (Table 1).

Exclusion criteria included chromosomopathies, major congenital malformations, profound resuscitation with chest compression and/or medication, parental history of immunodeficiency, or congenital infections.

### 2.2. RNA Extraction

Venous blood (0.5 mL) was obtained before the initiation of antibiotics both from cases and matched controls and mixed with 1 mL of RNA stabilizing solution (Tempus^TM^ Blood RNA tubes, Applied Biosystems^®^, Foster City, CA, USA) and stored at −20 °C until further processing. Total RNA was isolated using the MagMAX RNA isolation kit (Ambion/Applied Biosystems, Foster City, CA, USA) according to the manufacturer’s specifications.

### 2.3. Small Non-Coding RNAs and mRNA Expression Profiling

Small non-coding RNA expression profiling was performed using GeneChip miRNA 4.0 Array (Thermo Fisher Scientific, Waltham, MA, USA). The array contained 30,434 mature microRNA sequences from the miRBASE (v20) encoded miRNA coverage of 203 organisms, 2578 human mature miRNAs, and 1908 human snoRNAs and scaRNAs probe sets. Microarray experiments were conducted according to the manufacturer’s instructions. Briefly, 300 ng total RNA was labeled with FlashTag Biotin HSR RNA Labeling Kit (Thermo Fisher Scientific). The labeling reaction was hybridized on the miRNA array in hybridization oven 645 at 48 °C for 18 h. The arrays were stained with Fluidics Station 450 using fluidics script FS450_0002 and then scanned on GeneChip Scanner 3000 7G (Thermo Fisher Scientific).

mRNA expression profiling was performed using GeneChip Human Gene 1.0 ST Array (Thermo Fisher Scientific). The array comprised more than 750,000 unique 25-mer oligonucleotide features constituting 33,297 well-annotated genes. Then, 300 ng total RNA was labeled and hybridized on the hybridization oven 640 at 45 °C for 16 h. The arrays were stained with Fluidics Station 450 and then scanned on GeneChip Scanner 3000 7G (Thermo Fisher Scientific).

### 2.4. Data Analysis of Microarrays

Data (.CEL files) were analyzed and statistically filtered using software Partek Genomic Suite 6.6 (Partek Inc., St. Louis, MO, USA). Input files were normalized with the RMA algorithm for gene array on core meta probe sets or miRNAs. A one-way ANOVA was performed with the Partek Genomics Suite across all samples. Statistically significant small non-coding RNAs and mRNAs between different groups studied were identified using a model analysis of variance of *p*-value ≤ 0.01 and FDR ≤ 0.05, respectively. The imported data were analyzed by principal components analysis to determine the significant sources of variability in the data.

Non-supervised gene set enrichment analysis (GSEA) was carried out by obtaining the target genes from the 33 miRNAs using a computational analysis from the information allocated in the following databases: TargetScan (conserved site context scores, version 7.1), miRDB (release 5.0) and validated information from miRTarBase (version 7.0). The analysis was based in a prediction blast with an e-value of 10^−5^ and a weight of 0.9. GSEA was performed by ranking all target genes based on the fold-change values of each individual miRNA.

Finally, the most specific enrichment analysis was done by selecting differential expressed miRNAs and mRNAs in septic group versus control neonates. These miRNAs and genes were imported into Pathway Studio v12 (Pathway Studio^®^ software, Elsevier^®^ Inc., Rockville, MD, USA) to classify the relevant biological processes and subnetworks groups.

### 2.5. Data Analysis of Clinical and Demographic Analysis

Categorical variables were compared using χ2 or Fisher’s exact test (two-tailed). Continuous variables were expressed as mean ± SD or medians with interquartile range depending on data distribution. Two-tailed Student’s *t*- or Mann–Whitney U-tests and analysis of variance (ANOVA) or Kruskal–Wallis were used to compare 2 or 0.2 groups as appropriate. Kolmogorov–Smirnov analysis was performed to test the normal distribution of the data. Data analysis was performed by using SPSS version 17.0 (SPSS Inc., Chicago, IL, USA). Significance was considered for *p* ≤ 0.05.

## 3. Results

### 3.1. Patients’ Characteristics

No statistically significant demographic and clinical differences were observed in VLBW with late-onset Gram-positive sepsis in comparison with control neonates (Table 2). The 11 isolated Gram-positive bacteria were *Streptococcus coagulase negative* (n = 8), *Enterococcus faecalis* (n = 2), and *Staphylococcus aureus* (n = 1).

### 3.2. Principal Component Analysis (PCA) of Neonatal miRNome

**Figure 1:**
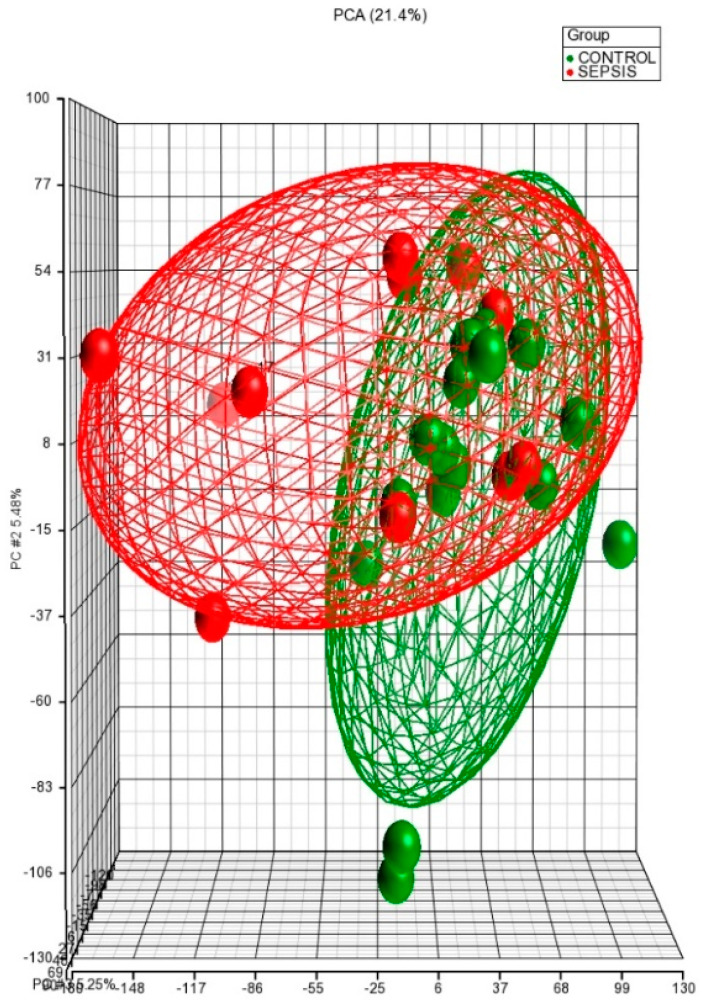
Principal component analysis (PCA) based on the overall miRNome. Individual neonates are plotted based on their respective positions along the three axes: 16 controls in green and 11 Gram-positive bacteria in red.

The tridimensional PCA from 27 neonates of whole miRNome (11 Gram-positive bacteria in red and 16 controls in green) identified two well-defined groups (Figure 1). The percentage of variability found was 21.4%. This result shows that miRNome clearly discriminates between Gram-positive sepsis and control samples.

### 3.3. miRNome Differential Analysis

The one-way ANOVA test was performed on Gram-positive septic neonates and controls. This analysis identified 217 differentially expressed miRNAs (*p* value < 0.01); 168 miRNAs were overexpressed (77.42%) and 49 underexpressed (22.58%) in the septic group versus the control group.

**Figure 2:**
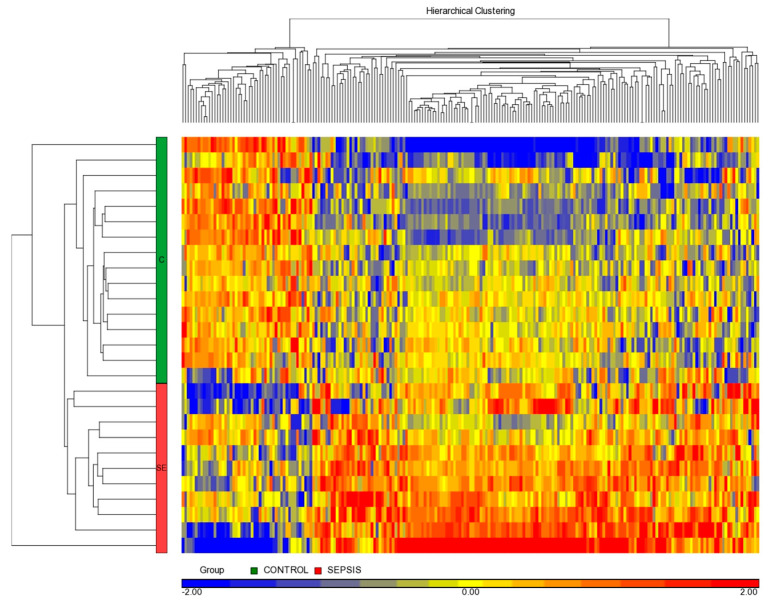
Heatmap of 217 differentially expressed miRNAs. Each column represents an miRNA and each line a neonate. Overexpressed miRNAs are represented in red and under-expressed miRNAs in blue. The left green bar represents control neonates (n = 16) and the left red bar is Gram-positive septic neonates (n = 11).

The unsupervised hierarchical clustering of differential miRNAs confirmed two clearly defined groups of patients (Figure 2) in terms of the septic or non-septic group.

### 3.4. Combined Analysis: miRNAs Susceptible to Regulate mRNAs in Gram-Positive Sepsis versus Controls Neonates

We identified the transcriptomic profile using same samples as before with all miRNAs. We distinguished 4297 differential expression genes (FDR < 0.05) using one-way ANOVA.

We combined 217 differentially expressed miRNAs with 4297 differentially expressed genes to evaluate the quantity of potential miRNAs that could regulate these mRNAs. The result was 33 miRNAs (see Table 3).

This combined study reduces the number of miRNAs that may be more relevant as upstream targets from 217 to the 33. These miRNAs could be considered regulators of the most important genes in the septic process.

The number of genes associated with each miRNAs was as follows: miR-15a-5p (567 genes), miR-30c-5p (534 genes), miR-30b-5p (534 genes), miR-27b-3p (500 genes), and miR-23b-3p (493 genes). Other miRNAs such as miR-93-5p, miR-20b-5p, miR-20a-5p, miR-17-5p, miR-106b-5p, and miR-106a-5p could regulate 487 genes.

A total of 3706 genes seem to be regulated by the 33 miRNAs selected. The unsupervised gene set enrichment analysis revealed that the main significant biological processes involved in LOS correspond to the immune and inflammatory responses and the development of the vascular system.

Nevertheless, most specific processes associated with these main groups may appear, when the enrichment analysis is done using only those genes reported in a previous sepsis study, to be controlled by the 33 miRNAs of interest.

### 3.5. Biological Analysis from a Combined Analysis

Relevant biological processes related to the immune system and the inflammatory response are summarized in Table 4.

Furthermore, we obtained main nodes or master regulators with a subnetwork analysis (see Table 5).

**Figure 3:**
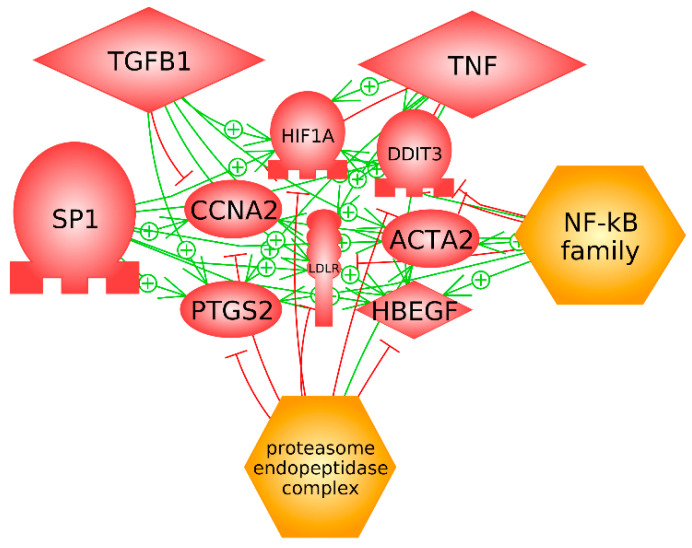
Connections between seven overlapping genes (CCNA2, HIF1A, DDIT3, ACTA2, HBEGF, LDLR, and PTGS2) in the top five master regulators (SP1, TGFB1, TNF, NF-kB family, and proteasome endopeptidase complex). The line in green is positive effect and the line in red is negative effect.

We detected seven overexpressed genes that were in common in these top five master regulators (Figure 3): Cyclin A2 (CCNA2) (*p*-value 0.0184), human hypoxia inducible factor 1, alpha subunit (HIF1A) (*p*-value 0.0033), DNA damage inducible transcript 3 (DDIT3) (*p*-value 0.0323), actin alpha 2, smooth muscle (ACTA2) (*p*-value 0.0446), heparin binding EGF like growth factor (HBEGF) (*p*-value 0.0027), low density lipoprotein receptor (LDLR) (*p*-value 0.0003), and prostaglandin-endoperoxide synthase 2 (PTGS2, alias COX-2) (*p*-value 0.0073).

Surprisingly, we identified only one differential miRNA expression, miR-17-5p (Biological *p*-value of 0.0015), of a total of 33 miRNAs, that regulated a higher number of these genes (487 differential expression genes, see Figure S1), specifically: CCNA2, HIF1A, ACTA2, LDLR, and PTGS2.

## 4. Discussion

Identifying differentially expressed miRNAs could help in establishing a sensitive, specific, and rapid diagnostic method to improve diagnosis and treatment and, subsequently, survival in neonatal sepsis [26].

We identified, for the first time, the miRNomic signature in VLBW neonates with LOS due to Gram-positive bacteria. Our results showed 217 differentially expressed miRNAs, of which 33 were candidates to regulate the transcriptome in Gram-positive sepsis neonates in a combined analysis. Our biological and sub-networks’ analysis confirmed that these miRNAs have a noteworthy role in immune and inflammatory response regulation.

Many miRNAs have been identified as important modulators of the immune response. miR-146b and miR-27b inhibit pro-inflammatory cytokines’ secretion, while miR-146a is induced by *NF-κB* and modulates *IFN*γ signaling pathway in T regulatory cells [27,28,29]. Both miR-106b and miR-17 are associated with TFG-signaling [30]. miR-20a is reduced after mycobacterial infection [31]. Moreover, miR-326 promotes Th17 cells and miR-31 negatively regulates peripherally derived regulatory T-cell generation [32]. miR-107 maintains the intestinal microbiota to improve immunity and miR-106a regulates Toll-like receptor 4 expression in lipopolysaccharide (LPS)-mediated immune response [33,34]. Furthermore, miR-106a and miR-140 regulate TNF alpha and IL-10, and miR-152 contributes to immune homeostasis [35,36].

We have highlighted both miR-15a and miR-23 because they regulate the most genes of our transcriptome amounting 597 and 493 genes, respectively (see Figure S1). miR-23 is an important regulator of the innate immune response and several inflammatory processes by targeting metalloproteinase 10 and mediating in the development of myocardial dysfunction [37]. Both have been previously described in full-term neonates with sepsis as potential clinical biomarkers that reflect the disease; nevertheless, they have not been evaluated in VLBW infants [19,38].

We found an under-expression of miR-23b coinciding to Fatmi et al. [38], who reported increased levels of miR-23b in EOS, but decreased levels in LOS. Unlike Wang et al. [19], who reported miR-15a over-expression in term neonates with LOS, we found an under-expression. Of note, our population was composed of VLBW infants that could show an immature response. Moreover, miR-15a down-regulates the expression level of *TLR4* and *IRAK1* induced by LPS, which have been more commonly related to Gram-negative sepsis, while our sepsis was all caused by Gram-positive bacteria [19].

In addition, the under-expression of miR-17 that regulates a total of 487 differential expression genes (see Figure S1) in neonates with Gram-positive LOS compromises vital processes such as cell proliferation, cell viability, T-cell function, cell survival, cell cycle progression, and immune response [39,40,41,42].

We have focused on a very specific type of sepsis to obtain a homogenous signature as different transcriptomic profiles have been described between Gram-positive and Gram-negative neonatal sepsis [43] This could explain why we have not obtained the same significant miRNA profile as previous studies that focused on different types of sepsis or in those that explored the regulatory role of miRNA in the LPS-induced inflammatory response, which is more characteristic of Gram-negative sepsis [23,44].

Our study has some limitations. First, as it was a pilot study with a small number of patients, we did not validate our results in an independent cohort. The subnetworks regulated by the most significant miRNAs are in concordance with those obtained in other transcriptomic studies previously performed in this population [45,46,47].

Our findings suggest that specific miRNAs discriminate between VLBW infants with LOS from non-septic controls and may be potential therapeutic targets for infections caused by Gram-positive bacteria through the modulation of the neonatal immune system.

## Figures and Tables

**Table 1:** Risk factors and clinical signs of sepsis.

**Risk Factors of Sepsis**
Maternal chorioamnionitis diagnosed by culture and clinical sympthoms. Babies born to mothers with group B *Streptococci* (GBS) isolation in urine, rectal, and/or vaginal swabs when mother received no completed antibiotic treatment (at least 2 doses of Ampiciline before labor). Newborns of mothers non-tested for GBS having any of these risk factors: - Premature rupture of membranes >18 h - Presence of fever during labor - Premature labor no completely treated with antibiotics. Neonates carrying any of these devices at least 24 h prior to symptoms: peripheral or central line, ventriculoperitoneal shunting valve, endotracheal tube or tracheostomy, thoracic drainage, uretral catheter. Neonates who had undergone surgery in the 72 h prior to symptoms.
**Clinical Signs of Sepsis**
Temperature instability: Rectal temperature ≥38 °C or ≤36 °C. Respiratory symptoms: respiratory distress, apnoea, or cyanosis. Cardiovascular symptoms: hypotension (blood pressure < 5th percentile for age), tachycardia (HR > 180 min), bradycardia (HR < 100 min), or poor perfusion. Neurological symptoms: clinical or electrical seizures, hypotonia, or lethargy. Gastrointestinal symptoms: vomiting, poor feeding or feeding intolerance, and/or abdominal distension.

**Table 2:** Demographic and clinical characteristics of very low birth-weight (VLBW) infants with Gram-positive sepsis (n = 11) and healthy controls (n = 16).

	Gram-Positive Sepsis (n = 11)	Non-Septic Controls (n = 16)	*p*-Value
Gestational age (weeks)	26 (26–29)	29 (27–30)	0.22 ^a^
Gender: male	5 (45.5)	10 (62.5)	0.38 ^b^
Birth weight (g)	940 (868–1175)	1270 (879–1445)	0.29 ^a^
Ethnicity: Caucasian	7 (63.6)	15 (93.7)	0.12 ^b^
Delivery:			0.053 ^b^
Vaginal	6 (54.5)	3 (18.7)	
C-Section	5 (45.5)	13 (81.2)	
Apgar 1 min	8 (5–9)	6 (5–7)	0.18 ^a^
Apgar 5 min	9 (9–10)	9 (8–9)	0.63 ^a^
Nutrition: Breastmilk	11 (100)	15 (93.7)	1 ^b^
Central line	8 (72.7)	8 (50)	0.24 ^b^
Days after birth at sample collection	11 (7–19)	14 (3–26)	0.72 ^a^
Weight at sample collection (g)	1020 (875–1220)	1210 (888–1473)	0.23 ^a^

(^a^) Student’s *t*-test, (^b^) Fisher’s exact test (two-tailed). Continuous variables are expressed in median (interquartile range). Categorical variables are expressed in n (%).

**Table 3:** Thirty-three miRNAs obtained after the analysis combined with the 217 miRNAs and the 4297 genes filtered by *p*-value.

Transcript ID (*Array* Design)	*p*-Value (SEPSIS vs. CONTROL)	Fold Change (SEPSIS vs. CONTROL)
hsa-miR-31-5p	0.0017	−3.05
hsa-miR-1271-5p	0.0089	−2.77
hsa-miR-326	0.0013	−2.39
hsa-miR-146b-5p	0.0050	−2.38
hsa-miR-140-5p	0.0091	−2.26
hsa-miR-409-5p	0.0084	−2.24
hsa-miR-668-3p	0.0038	−2.21
hsa-miR-27b-3p	0.0028	−2.12
hsa-miR-28-5p	0.0019	−2.06
hsa-miR-152-3p	0.0080	−1.95
hsa-miR-431-5p	0.0094	−1.95
hsa-miR-106b-5p	0.0063	−1.73
hsa-miR-151a-3p	0.0002	−1.72
hsa-miR-15a-5p	0.0046	−1.71
hsa-miR-339-5p	0.0068	−1.70
hsa-miR-30b-5p	0.0079	−1.68
hsa-miR-146a-5p	0.0024	−1.68
hsa-miR-20a-5p	0.0008	−1.64
hsa-miR-20b-5p	0.0005	−1.60
hsa-miR-532-5p	0.0059	−1.51
hsa-miR-30c-5p	0.0044	−1.47
hsa-miR-106a-5p	0.0006	−1.43
hsa-miR-17-5p	0.0007	−1.39
hsa-miR-23b-3p	0.0017	−1.37
hsa-miR-93-5p	0.0088	−1.22
hsa-miR-425-5p	0.0083	−1.21
hsa-miR-1298-5p	0.0054	−1.19
hsa-miR-107	0.0009	−1.14
hsa-miR-103a-3p	0.0004	−1.14
hsa-miR-372-3p	0.0054	1.11
hsa-miR-760	0.0063	1.34
hsa-miR-6088	0.0011	1.68
hsa-let-7c-5p	0.0089	2.09

**Table 4:** The most relevant biological processes filtered by enrichment *p*-value.

Biological Processes	*p*-Value	Number of Genes
Treg-Cell Differentiation	4.92 × 10^−9^	27
Th2-Cell Differentiation	1.84 × 10^−8^	26
Nociception Expression Targets Signaling	5.62 × 10^−8^	43
mTOR Signaling	2.80 × 10^−7^	42
Serotonin Receptors Signaling	3.06 × 10^−7^	23
Catecholamines Secretion from Adrenal Gland	5.00 × 10^−7^	26
Peripheral T-Cell Tolerance	6.81 × 10^−7^	23
Vascular Motility	7.59 × 10^−7^	28
Th1-Cell Differentiation	1.06 × 10^−6^	24
Thrombopoietin Receptors Signaling in Platelet Maturation	1.71 × 10^−6^	16

**Table 5:** The most relevant subnetworks filtered by enrichment *p*-value.

Subnetworks	*p*-Value	Number of Genes
Protein targets of proteasome endopeptidase complex	1.4 × 10^−213^	266
Protein targets of TNF	5.6 × 10^−213^	315
Protein targets of TGFB1	4.4 × 10^−212^	308
Protein targets of SP1	5.7 × 10^−193^	263
Protein targets of NF-kB family	4.8 × 10^−171^	247
Protein targets of mitogen-activated protein kinase	2.2 × 10^−154^	205
Protein targets of TP53	3.3 × 10^−145^	200
Protein targets of ubiquitin	1.2 × 10^−139^	172
Protein targets of INS	1.0 × 10^−134^	193
Protein targets of MAPK1	1.5 × 10^−134^	186

## Supplementary Materials

The following are available online at https://www.mdpi.com/article/10.3390/diagnostics11081389/s1, Figure S1: Number of genes that are regulated for each miRNA in the combined study.
