# Supplementary figures and images for: miRNomic Signature in Very Low Birth-Weight Neonates Discriminates Late-Onset Gram-Positive Sepsis from Controls
Source: Diagnostics (Basel). 2021 Jul 31;11(8):1389. doi: 10.3390/diagnostics11081389 (PMC8391178; doi:10.3390/diagnostics11081389)

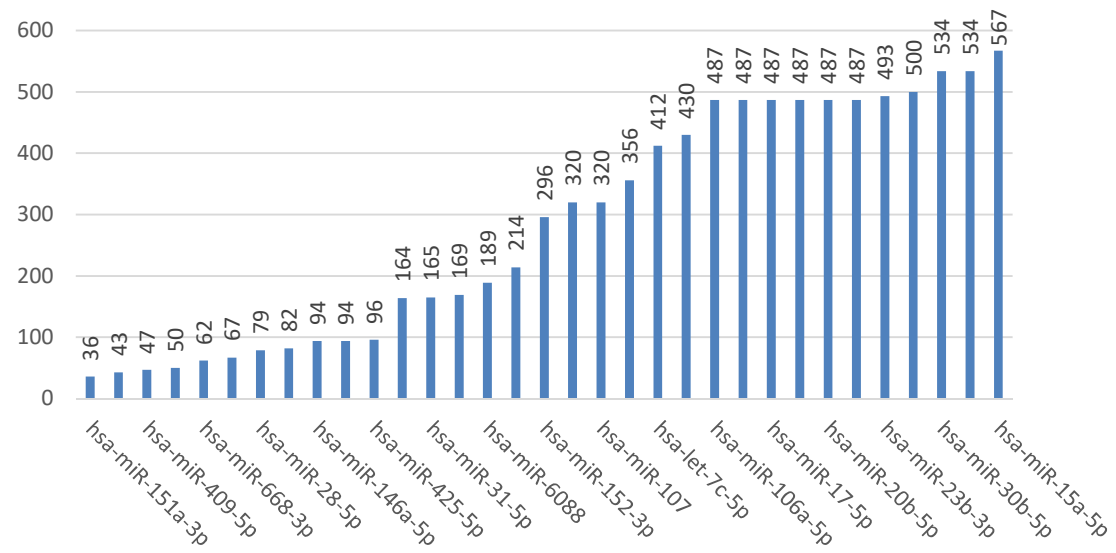

**Figure S1.** Number of genes which are regulated for each miRNA in the combined study.

Supplement: Supplementary file 1 [file diagnostics-11-01389-s001.zip › diagnostics-1255896-supplementary.pdf]
